# Serially mediated effects of psychological inflexibility on quality of life of refugees in Uganda during coronavirus pandemic

**DOI:** 10.1371/journal.pgph.0002450

**Published:** 2023-10-25

**Authors:** Martin Mabunda Baluku, Stewart Ssebagala, Hilary Mugabo Mukula, Khamisi Musanje

**Affiliations:** School of Psychology, Makerere University, Kampala, Uganda; Georgetown University, UNITED STATES

## Abstract

The unexpected outbreak and rapid spread of COVID-19 necessitated radical and stringent control measures, consequently changing how people live globally. To vulnerable populations like refugees, who were already living a disrupted life, the outbreak of COVID-19 and accompanying control measures complicated their living conditions and drastically affected their mental health and, consequently, their quality of life. The current study aimed to test whether psychological inflexibility was a factor in lowering the quality of life of refugees in Uganda during the COVID-19 pandemic. The study further examines whether the effects of psychological inflexibility on quality of life were serially mediated by avoidance coping, perceived threat, adherence to COVID-19 control measures, and general mental health states. The study was conducted among refugees living in Kampala city suburbs and Bidibidi refugee settlement in Uganda. Data was collected using a survey questionnaire during the partial reopening of the economy in mid-2020, after the first lockdown. The analysis assesses a serial mediation model of the effects of psychological inflexibility on the quality of life of refugees through avoidance coping, perceived threat, adherence to COVID-19 control measures, and mental health using PROCESS Macro. The study involved 353 participants. Our analyses revealed that psychological inflexibility was negatively associated with the perceived threat, adherence, and quality of life. Psychological inflexibility was positively associated with avoidance coping and poor mental health. The data supported all hypothesized mediation paths. These findings support literature suggesting that psychological inflexibility is a maladaptive attribute that thwarts positive coping and behavior adjustment in times of crisis. Consequently, psychological inflexibility can worsen mental health problems and quality of life, especially in populations such as refugees in low-income countries who live in precarious conditions. Incorporating interventions that reduce psychological inflexibility in crisis management efforts can help refugees maintain good psychological functioning and quality of life.

## Introduction

The coronavirus outbreak was first confirmed in Wuhan, China, at the end of 2019. With the virus rapidly spreading to all the continents within five months, with over five (5) million confirmed cases and over 300000 deaths [1], radical and stringent measures to control its spread were required. However, these changed how people live [2], and some of such changes will likely stay for a long time. For example, social distancing, stay at home, self-isolation or quarantine, curfews, economic lockdown, and hygiene measures have not only resulted in lifestyle changes but also have had consequences for mental health and Quality of Life “QoL” [1, 3, 4].

The situation could even have been worse for refugees in low-income contexts who experienced several challenges during the pandemic, including limited access to health care, low social support, crowding, poor sanitation, and barriers to communication and movement [5–7]. Therefore, they were more likely to report poor mental health and QoL during the pandemic than other populations [3, 7]. In addition, refugees are among the low-income and discriminated minorities who are often excluded from welfare programs, experience xenophobia and stigmatization, and face the risk of arrest and deportation [5, 8, 9]. These realities increase their vulnerability to contagion and the adverse outcome of the pandemic. The situation of refugees in Uganda was not helped by the fact that the first confirmed cases were of people entering the country. Moreover, with the porousness of Uganda’s borders and the mobility nature of refugees, they were not only at risk of contracting the virus but were also perceived as coronavirus transmitters [10].

Uganda hosts over 1.5 million refugees mostly from a significant number of refugees from South Sudan (57.1%), Somalia (4.1%), and the Democratic Republic of Congo (32%) [11]. These refugees live in congested suburbs of towns and rural settlements with crowded makeshift houses, which increases the risk of contagion [12]. The limited resources imply that refugees scramble for and have to queue for long hours to access social and basic goods such as water and health care [13]. Refugees mainly survive on food rations and humanitarian aid from international actors such as the World Food Programme, which they supplement with small business ventures [10, 14], which were affected by the lockdown of the economy. Whereas COVID generally impacted food security and increased poverty, the impact among refugee populations [15], especially those in Uganda, has worsened since they now face malnutrition challenges [16]. An essential component of responses to crises is adequate community education and timely communication, which helps to dispel fears and ambiguity and rallies individuals to gear efforts toward tackling health threats [17, 18]. This is essential to buffering against mental health challenges associated with pandemics and critical for improving adherence to control measures. However, refugees face the language barrier challenge and thus often feel left out on critical communications about the pandemic [19, 20], which might have exacerbated the feeling of discrimination, given that COVID-19 also led to a rise in cases of xenophobia and social stigmatization [9, 21, 22]. All these issues directly affect the psychological health and QoL of refugees, who were already suffering from pre and post-migration stress associated with settling in the host community [23].

Health epidemics and pandemics generally negatively affect mental health and quality of life [21]. Whereas catastrophic life events such as COVID-19 evoke general reactions and experiences across populations; thus diminishing human functioning [24], individual psychological attributes and resources influence the magnitude of psychological outcomes people encounter during such events. The present study focuses on psychological inflexibility as a maladaptive attribute that could have negatively affected the QoL of refugees during COVID-19 through avoidance coping. Substantial research suggests that psychological inflexibility is strongly associated with psychological problems [25–28]. However, research on how psychological inflexibility influences QoL is still underdeveloped [28]. Psychological inflexibility represents the inability to accept and to be in contact with one’s difficult inner experiences [28, 29], consequently suppressing or avoiding such experiences [29, 30]. However, the attempts to suppress unpleasant experiences only result in a paradoxical increase of the same experiences [31]. Hence, psychologically inflexible individuals are more likely to distract or avoid unpleasant experiences when faced with adversity, compromising their mental health and QoL [32–34].

**Fig 1:**
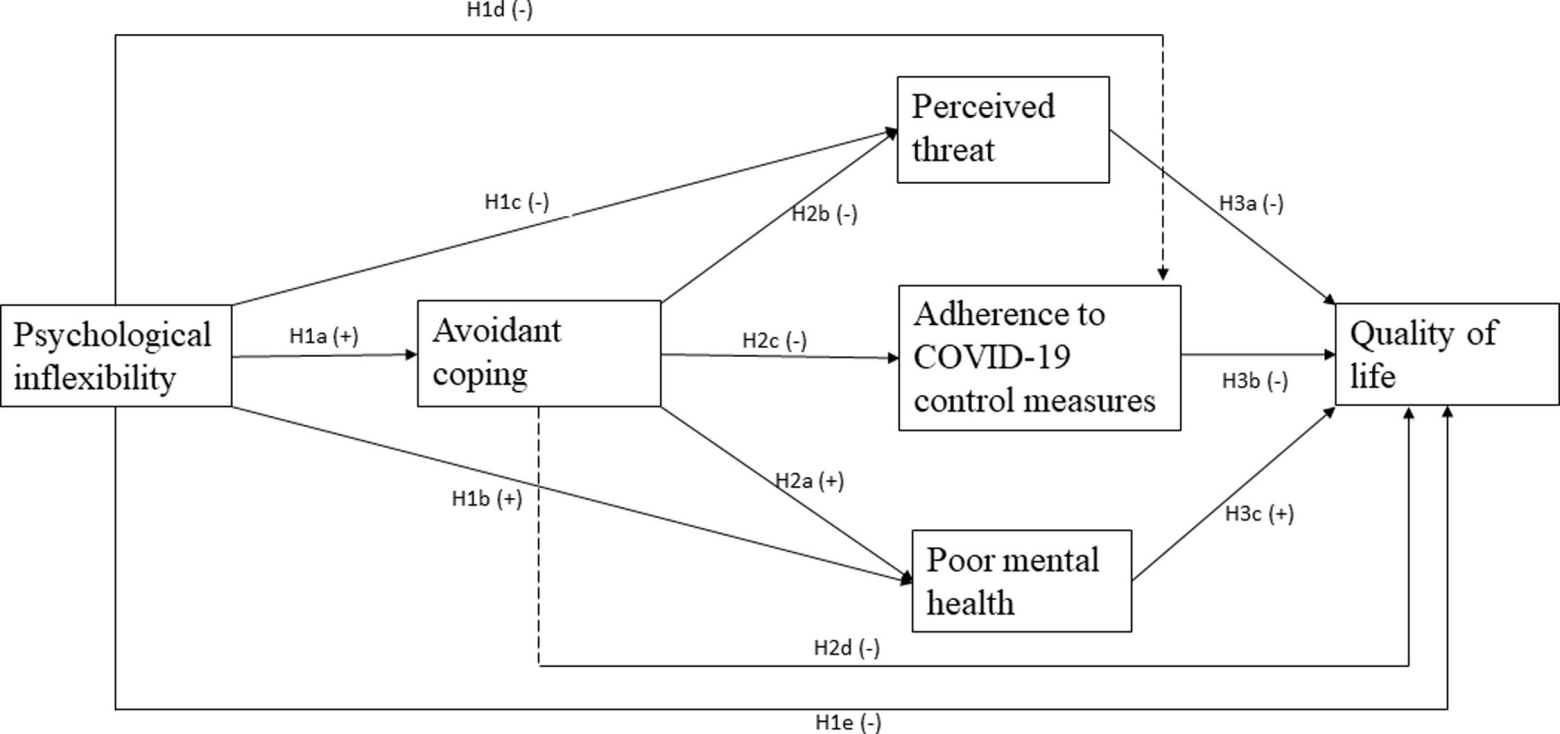
Conceptual model.

Adherence to COVID-19 control measures meant individuals were required to make sudden changes to their social behavior and activities. This implies that the limitations imposed during the COVID-19 pandemic and the efforts to adhere to them were likely to cause anxiety, stress, and depression [1, 35], indicating poor mental health and lower QoL. Psychological inflexibility and avoidance coping have connotations for perceiving the threat a situation poses. Yet the perceived threat level has implications for distress, wellbeing, and adherence to control measures [36–39]. As depicted in Fig 1, we propose a serial mediation of the effects of psychological inflexibility on the QoL of refugees in Uganda during COVID-19 through avoidant coping, which could have affected perceptions of the threat posed by the COVID-19 situation, adherence to control measures, and mental health; and consequently, impacting on QoL.

### Theory and hypothesis development

Psychological inflexibility, which concerns an individual’s inability to be in contact with and accept difficult internal experiences [24, 40], is one of the central constructs in the Acceptance and Commitment Therapy “ACT” [30, 31, 41]. In the ACT model, psychological inflexibility represents rigidity in reactions to difficult or unwanted inner experiences, including negative thoughts, memories, and physiological sensations [29, 41–43]. Although there is limited empirical research explaining the association between psychological inflexibility and QoL, theoretical evidence suggests that psychological inflexibility could be detrimental to QoL [44]. In the ACT model, psychological inflexibility is conceptualized to inhibit behavior change in a situation that requires adjustment, consequently lowering the QoL [30, 41, 45]. The evasion of the negative internal experiences that characterize psychological inflexibility facilitates the development of psychopathology and pain [45]. In this direction, Peltz et al. (2020) show that psychological inflexibility is associated with lower functionality in some QoL aspects, including leisure, employment, and family [46]. In a study of pain and functioning among juveniles with idiopathic arthritis, psychological inflexibility was found to be associated with lower general QoL and health QoL [47]. Studies among refugee populations have so far applied psychological inflexibility to explaining mental health [25]. Currently, there is inadequate research linking psychological inflexibility to the QoL of refugees in different contexts. The present study provides evidence from a leading refugee hosting yet a low-income country in the context of the COVID-19 pandemic.

The current study proposes that the effects of psychological inflexibility on QoL occur in a serial mediation process, with avoidance coping as the primary mediator. Psychological inflexibility and its opposite, psychological flexibility, have been conceptualized as closely related to coping strategies [48, 49]. Psychological inflexibility is mainly associated with avoidance coping. Whereas flexibility is seen as essential in the adjustment and healing process [50], inflexibility, on the other hand, tends to obstruct meaningful coping and adjustment [28]. Experiential avoidance, which concerns the tendency to evade complex thoughts, feelings, and experiences, is one of the mechanisms through which psychological inflexibility is manifested [48, 51, 52]. Since avoidance coping does not target the threat [53], it is more likely to lead to maladaptive behavior and reduced QoL. Whereas approach coping is associated with an increase in wellbeing, avoidance coping, on the other hand, is associated with distress and unhappiness [50, 54].

The emergence of COVID-19 was associated with several psychological challenges. Notably, the nature of the disease, the wide publicity about infections, and the risk of death appeared frightening and could impact life negatively [55]. The closure of workplaces and social services such as educational institutions implied that people lost their daily routines. At the same time, there were several barriers to alternatives, such as working or studying online, increasing stress levels [56, 57]. For example, stay at home as a preventive measure meant that family members stayed together longer than usual. Whereas this improved bonding within families, it also resulted in psychosocial problems such as domestic and intimate partner violence and depression [58]. From the transactional stress model, the appraisal of the COVID-19 threat and coping have implications for stress, adherence, and wellbeing [37]. The perceived vulnerability to infection and the life changes associated with the disease influence the amount of felt threat [59, 60] and consequently the impact on mental health and wellbeing [38]. Whereas the subjective appraisal of the threat influences the coping strategy that individuals use [61], we propose that individuals with high psychological inflexibility are already inclined to adopt avoidance coping, which hinders their ability to adequately appraise the threat and adopt coping strategies that fit the nature and magnitude of the threat. Usually, appraisal of threat leads to behaviors geared towards threat reduction [62]. Concerning psychological inflexibility and avoidance coping, individuals may understate the threat to serve current behavior, creating short-term relief and long-term compromise of QoL. A recent study indicates that both avoidance and approach coping strategies were positively associated with quality of life of refugees in the early stages of the COVID-19 pandemic [63].

Behavior change and adherence to control measures depend on the magnitude of the perceived threat or vulnerability to contagion and on individual traits. The ACT model is a framework for behavior change [30, 41, 45] in which flexibility facilitates positive behavior change while inflexibility represents rigidity in behavioral adjustment [28]. The experiential avoidance mechanisms nested in inflexibility [27, 49, 51] imply that individuals with high psychological inflexibility were less likely to adhere to preventive measures, thus increased their vulnerability to contagion and mental health problems in the long term. Moreover, there is empirical evidence, albeit insufficient, that individuals reporting lower adherence to COVID-19 preventive measures were more likely to report dissatisfaction with several aspects of their QoL [64].

Since psychological inflexibility inhibits behavior adjustment through experiential avoidance, it is a risk factor for developing psychological problems [30, 65]. For example, psychological inflexibility has been linked to posttraumatic stress disorder symptoms in different populations, including veterans and refugees [25, 66, 67]. Moreover, the COVID-19 pandemic has generally exacerbated mental health problems in all populations across the globe [68], in addition to the already high levels of mental health problems that usually exist in refugee populations [69]. A decline in mental health can potentially lower QoL, especially for refugees with high levels of psychological inflexibility [48]. In this direction, extant empirical findings suggest that psychological inflexibility tends to aggravate the adverse effects of COVID-19 stress [67].

The present study aimed to explore the impact of psychological inflexibility on QoL through a serial-mediated model. Based on the above literature, we tested the following hypotheses.

Hypothesis 1. Psychological inflexibility is positively associated with (a) avoidance coping and (b) poor mental health, but negatively associated with (c) perceived threat of COVID-19, (d) adherence to COVID-19 control measures, and (e) the quality of life of refugees during the coronavirus pandemic.

Hypothesis 2. Avoidance coping is positively associated with (a) poor mental health and negatively associated with (b) perceived threat of COVID-19, (c) adherence to COVID-19 control measures, and (d) quality of life of refugees during the coronavirus pandemic.

Hypothesis 3. Quality of life is further influenced negatively by (a) perceived threat and (b) adherence to COVID-19 control measures, and (c) positively by mental health.

Hypothesis 4. The effects of psychological inflexibility on the quality of life of refugees during the coronavirus pandemic were mediated by (a) avoidance coping via (b) mental health, (c) the perceived threat of COVID-19, and (d) adherence to COVID-19 control measures.

## Methods

The paper uses data from a larger study, “Investigating and Addressing COVID-19 Related Mental Health Challenges in Refugee Settlements and Host Communities in Uganda,” to which the first author was the principal investigator. Two other studies have used the data set. The first examines the role of psychological capital and coping strategies in boosting refugees’ mental health and quality of life during the pandemic [63]. The second interrogates the moderating role of coping strategies in the association of psychological inflexibility with post-traumatic stress disorder and adherence to COVID-19 control measures [70]. The current study differs from these by highlighting the serial mediation process through which psychological inflexibility could have negatively affected the quality of life of refugees during the pandemic, with particular attention to avoidance coping, perceived threat, and lowered mental health as the mediating links.

### Ethics approval statement

This paper has been developed from the larger research project titled “Investigating and Addressing COVID-19 Related Mental Health Challenges in Refugee Settlements and Host Communities in Uganda”. The project was given ethical clearance by the Gulu University Research Ethics Committee, Clearance No. GUREC-2020-32.

### Participants

The study was conducted in two refugee settlements that differ in socio-economic contexts. These included Kampala metropolitan area and Bidibidi in the West Nile region. Bidibidi was preferred for rural refugees because it was the world’s second-largest refugee settlement at the time, hosting over 270,000 refugees [71]. In contrast, Kampala metropolitan area hosts the majority of urban refugees in Uganda, with a population of over 80,000 refugees. Refugees in rural settlements have access to humanitarian aid, albeit inadequate and have access to land for cultivation. On the other hand, urban refugees are fully responsible for their survival needs [10]. Most urban refugees depend on small-scale entrepreneurial activities, which were also grossly affected by COVID-19-imposed economic lockdowns. These differences in the contexts were deemed essential to perceptions of the threat of COVID-19, adherence to control measures, and pandemic-related mental health problems.

Overall, 402 refugees consented to participate (73.4% males; 57.4% from Bidibidi settlement). Based on the G-power v3.1 sample size calculator [72], the minimum sample recommended for the regression analysis model with ten (10) predictor variables, an anticipated effect size of 0.15, desired probability level of 0.01, and the desired statistical power of 0.99 is 279. Therefore, our sample size was adequate to achieve statistically satisfactory effect sizes. In addition, participants were relatively young refugees (M = 29.81 years, SD = 8.64, Range = 18–70 years), and the average period they had lived in Uganda was 4.65 years (SD = 3.45, Range = 1–25 years). Majority of the participants were refugees from South Sudan (81.9%). Other countries of origin included Somalia (16.1%), Sudan–North (1.4%), and Chad (.6%).

### Procedure

The study was conducted using self-administered questionnaires. Participants signed written informed consent forms and were also required to confirm that they were at least 18 years old at the time of the study. A group administration approach was applied. Small groups of refugees ranging from 10–20 people were brought together in available open spaces within the refugee settlements where standard operating procedures (e.g., washing hands with soap, social distancing among participants, and using sanitizers) could be easily implemented. Each participant completed the survey questionnaire independently. However, the research team including native speakers were present to clarify any issues raised by participants. Data was collected during the partial reopening of the economy in May ‐ July 2020, following a four-month economic lockdown.

The survey questionnaire was available in English, Somali, and Arabic languages. These languages are widely spoken in the two refugee settlements. Quality of translation into Somali and Arabic was ensured by using the back translation procedure [73]. Specifically, native speakers were hired from among the refugee populations to translate the questionnaire from English to Somali and Arabic (2 people for each language). Different individuals were hired to translate back to English. The translators then worked together to resolve any differences observed during the back translation process.

### Measures

#### Outcome measure

*Quality of life*. We used the Short Form of the Quality of Life Enjoyment and Satisfaction Questionnaire “Q-LES-Q–SF” [74, 75], which is available from PhenX Toolkit (https://www.phenxtoolkit.org/protocols/view/180302). We preferred the short form of the Q-LES-Q because it comprises fewer items (14), hence ideal for a quick survey. In the current study, we excluded items 3, 12, and 13 because they focus on the enjoyment and satisfaction with work and medication [75], hence deemed not relevant to the context of refugees during the COVID-19 pandemic. Participants were asked to indicate the level of satisfaction with different aspects of their lives on a 6-point scale ranging from 1 (not at all satisfied) to 6 (very satisfied). The questionnaire showed appropriate internal consistency for the present study (α = .82).

#### Mediators

*Avoidance coping*. We used the Brief COPE [76] to measure avoidance coping. The scale comprises 28 self-report items that assess an individual’s coping styles in response to stressful experiences. The study focuses on the avoidance dimension. This aligns with the two-factor structure model comprising avoidant and approach coping [48, 77]. Avoidant coping was assessed using 12 items that focus on avoidance strategies, including self-distraction, denial, substance use, behavioral disengagement, venting, and self-blaming. Items were rated on a 6-point scale ranging from 1 (Not at all) to 6 (a lot). The instructions to respondents were adapted to the COVID-19 context: "indicate the degree to which you have engaged in each of the following behaviors since the outbreak of COVID-19”. A sample item for avoidant coping is “I’ve been using alcohol or other drugs to help me get through it.” The scale has been widely used to assess coping strategies in different contexts. In the present study, the scale showed appropriate internal consistency for avoidance coping (α = .70).

*Perceived threat of COVID-19*. We measured the perceived threat of COVID-19 using three (3) items explicitly developed for this study. The items included (1) COVID-19 does not exist, (2) there is no COVID-19 in Uganda, and (3) COVID-19 does not affect the people of my age group. These items are scored on a 6-point Likert scale ranging from 1 (totally disagree) to 6 (totally agree). These items were reverse coded so that high scores indicated high perceived threat levels. The items showed appropriate reliability (α = .70).

*Mental health*. We assessed the general mental health of refugees using the General Health Questionnaire (GHQ-28), which assesses the distress and wellbeing of an individual [78, 79]. It is a screening tool that was designed to detect possible psychiatric problems. The GHQ measures four aspects of mental health including somatic symptoms, insomnia, social dysfunction, and severe depression. In the present study, the 28-item version was used. The items were assessed on a 6-point scale ranging from 1 (never) to 6 (very often). Reliability analysis showed appropriate internal consistency (α = .81).

*Adherence to COVID-19 control measures*. We developed a measure to assess adherence to COVID-19 control restrictions [80] following the structure of the Morisky Medical adherence questionnaire [81]. The questionnaire assesses the level of adherence to COVID-19 control measures, including frequent handwashing with soap, using sanitizers, wearing facemasks, social distancing, and self-isolation. The questionnaire comprised 23 items rated on a 6-point scale ranging from 1 (never) to 6 (very often). A sample item is “how often do you forget to wear a face mask”. The questionnaire had appropriate internal consistency (α = .91).

**Predictor variable. *Psychological Inflexibility*** was measured using the Avoidance and Action Questionnaire “AAQ” [29], which assesses the rigidity in handling unpleasant internal events [82]. The AAQ comprises seven (items) that were rated on a 6-point scale ranging from 1 (not at all) to 6 (very much). A sample item is “I worry about not being able to control my worries and feelings”. We found appropriate reliability in the present study (α = .77).

### Analytic approach

The study aimed to assess how psychological inflexibility affected the QoL of refugees in Uganda during the coronavirus pandemic. To achieve this goal, we proposed a serial mediation model such that the effects of psychological inflexibility on quality of life are mediated by avoidance coping and further by the level of mental health, the threat of COVID-19, and the burden of adherence to COVID-19 control measures. To test this model, we conducted a serial mediation analysis in PROCESS Macro for SPSS v3.4 model 81 [83], which simultaneously tests for the effects of multiple mediators. Bootstrapping at 5,000 and confidence intervals at 95% were applied. Since sex, age, type of settlement, and the number of years lived in Uganda were related to some variables, they were added to the regression model as control variables.

## Results

Table 1 presents descriptive statistics, internal consistency (Cronbach’s α) coefficients, and correlations among the focal variables in the study. Concerning the control variables, regression findings in Table 2 show that females were more likely use of avoidance coping (*B* = .32, *p* < .01), low perceived thread of COVID-19 (*B* = -.44, *p* < .05), and low quality of life (*B* = -.30, *p* < .05). Regarding type of settlement, refugees in rural settlements were more likely to report using avoidance coping (*B* = .31, *p* < .01) and low perceived threat of COVID-19 (*B* = .41, *p* < .05).

**Table 1:** Correlations and descriptive statistics.

Variables	1	2	3	4	5	6	7	8	9	10
1. Gender^a^										
2. Age	-.01									
3. Type of settlement^b^	.17^**^	.09								
4. Years lived in Uganda	.09	.04	-.12^*^							
5. Psychological inflexibility	.09	.07	.06	.06						
6. Avoidant coping	.20^***^	.06	.20^***^	.03	.44^***^					
7. Poor mental health	.13^*^	.06	.04	-.04	.43^***^	.45^***^				
8. Perceived threat	-.17^**^	-.09	.03	-.04	-.32^***^	-.38^***^	-.22^***^			
9. Adherence to COVID control measures	-.16^***^	-.03	-.09	-.04	-.36^***^	-.37^***^	-.22^***^	.33^***^		
10. Quality of life	.14^*^	.07	.01	.01	-.11^*^	.15^**^	-.24^***^	-.20^***^	-.20^***^	
Mean		29.81		4.65	3.57	3.44	3.11	4.26	3.25	3.84
SD		8.64		3.45	1.40	1.02	.90	1.66	1.29	1.27
α					.78	.70	.81	.70	.91	.82

Note:

^*^p < 0.05

^**^p < 0.01

^***^p < 0.001; N = 353

^a^Male = 0, Female = 1

^b^Urban = 0, Rural = 1

**Table 2:** Serial mediation of effects of psychological capital on quality of life of refugees during the coronavirus pandemic.

Predictors	Avoidance coping			Poor mental health			Perceived threat of Cov-19			Adherence			Quality of life		
	*B*	*95% CI*		*B*	*95% CI*		*B*	*95% CI*		*B*	*95% CI*		*B*	*95% CI*	
		*LLCI*	*ULCI*		*LLCI*	*ULCI*		*LLCI*	*ULCI*		*LLCI*	*ULCI*		*LLCI*	*ULCI*
Gender^a^	.32^**^	.10	.53	.12	-.31	.07	-.44^*^	-.80	-.07	.24	-.53	.05	.30^*^	.02	.58
Age	.003	-.01	.01	.003	-.01	.01	-.01	-.03	.01	< .001	-.01	.02	.01	-.002	.03
Settlement type^b^	.31^**^	.12	.51	-.11	-.28	.06	.41^*^	.08	.74	-.04	-.30	.22	-.14	-.39	.11
Years lived in Uganda	.002	-.03	.03	-.02	-.05	.002	.002	-.04	.05	-.004	-.04	.03	-.01	-.05	.02
Psychological inflexibility	.30^***^	.23	.37	.19^***^	.12	.25	-.22^***^	-.34	-.09	-.22^***^	-.32	-.13	-.17^**^	-.27	-.07
Avoidance coping				.28^***^	.19	.38	-.49^***^	-.67	-.31	-.31^***^	-.45	-.17	.31^***^	.16	.46
Poor mental health													-.52^***^	-.67	-.37
Perceived threat of Cov-19													-.12^**^	-20	-.04
Adherence													-.19^***^	-29	-09
R^2^	.24^***^			.28^***^			.21^***^			.19^***^			.24^***^		

Note

^*^p < 0.05

^**^p < 0.01

^***^p < 0.001; N = 353

^a^Male = 0, Female = 1

^b^Urban = 0, Rural = 1

CI = Confidence Intervals (LLCI = lower limit confidence intervals, ULCI = upper limit confidence intervals)

This regression model shows the effects of psychological inflexibility and control variables on the mediators (avoidance coping, poor mental health, perceived threat, and adherence) and the outcome variable (quality of life). The model also simultaneously computed the effects of the mediators on quality of life.

As suggested in hypothesis 1, psychological inflexibility was positively associated with avoidance coping (*B* = .30, *p* < .001) and poor mental health (*B* = .19, *p* < .001); and negatively associated with the perceived threat of COVID-19 (*B* = -.22, *p* < .001), adherence to COVID-19 control measures (*B* = -.22, *p* < .001), and quality of life (*B* = -.17, *p* < .01). Hypothesis 2 was also supported since avoidance coping was positively related to poor mental health (*B* = .28, *p* < .001) and negatively associated with the perceived threat of COVID-19 (*B* = -.49, *p* < .001) and adherence to COVID-9 control measures (*B* = -.31, *p* < .001). Contrary to our assumption, avoidance coping was positively associated with quality of life (*B* = .31, *p* < .001); hence hypothesis 2d is not supported.

Concerning the mediators and in line with Hypothesis 3, the results in Table 2 further show that the second mediators, including mental health (*B* = -.52, *p* < .001), perceived threat to COVID-19 (*B* = -.12, *p* < .01), and adherence to COVID-19 control measures (*B* = -.19, *p* < .001) were negatively related to quality of life. Given that higher scores for mental health indicated poor mental health, this result implies that poor mental health was negatively associated with poor quality of life. All hypothesized mediation paths were supported, hence hypothesis 4 is supported.

The mediation indices in Table 3 show that the paths from psychological inflexibility to quality of life through avoidance coping (*B* = .09, *CI* [.04, 16]), mental health (*B* = -.10, *CI* [-.15, -.06]), perceived threat of COVID-19 (*B* = .03, *CI* [.01, .06]), and adherence to COVID-19 control measures (*B* = .04, *CI* [.01, .08]) were all significant. Similarly, the serial mediation hypotheses were supported. The paths from psychological inflexibility through avoidance coping and mental health (*B* = -.05, *CI* [-.07, -.02]), through avoidance coping and perceived threat of COVID-19 (*B* = .02, *CI* [.01, .04]), and through avoidance coping and adherence to COVID-19 control measures (*B* = .02, *CI* [.01, .03]) were significant. Overall, the regression models explained 24% of avoidance coping, 28% of mental health, 21% of the perceived threat of COVID-19, 19% of adherence to COVID-19 control measures, and 24% of the quality of life of refugees.

**Table 3:** Summary of direct and indirect effects of psychological inflexibility on quality of life.

Nature of effects	Effects	Boot CI	
		*LLCI*	*ULCI*
Total effects	-.11^*^	-.21	-.02
Direct effects	-.17^**^	-.27	-07
Total indirect effects	.06	-.02	.14
Psychological inflexibility → avoidance coping → QoL	.09	.04	.16
Psychological inflexibility → poor mental health → QoL	-.10	-.15	-.06
Psychological inflexibility → perceived threat → QoL	.03	.01	.06
Psychological inflexibility → adherence → QoL	.04	.01	.08
Psychological inflexibility → avoidance coping → poor mental health → QoL	-.05	-.07	-.02
Psychological inflexibility → avoidance coping → perceived threat → QoL	.02	.01	.04
Psychological inflexibility → avoidance coping → adherence → QoL	.02	.01	.03

Note

^*^p < 0.05

^**^p < 0.01

^***^p < 0.001; N = 353.

QoL = quality of life

CI = Confidence Intervals (LLCI = lower limit confidence intervals, ULCI = upper limit confidence intervals)

The arrows are indicative of the path direction.

## Discussion

Extant research evidence shows that the COVID-19 pandemic and the preventive measures affected how people live, impacting the different aspects of QoL across the globe [1, 2, 4, 68]. It has changed how people work and socialize with consequences for mental health and quality of life [21, 57, 84, 85]. Whereas highly distressing situations tend to impact people’s functioning across the board [24], there is evidence that responses to and outcomes of distressing events are also determined by individual attributes [36, 38, 86, 87]. The present study aimed to examine the effect of psychological inflexibility on the QoL of refugees in Uganda during the COVID-19 pandemic. The study demonstrates that the impact of psychological inflexibility on QoL occurred through a serial mediation process through avoidance coping, perceived threat posed by COVID-19, adherence to COVID-19 control measures, and general mental health.

The findings indicate that psychological inflexibility strongly negatively affected the QoL of refugees during the COVID-19 pandemic. This is consistent with previous research showing that psychological inflexibility is associated with a decline in functioning in the different domains of QoL in times of distress [46, 47, 85]. Theoretically, the avoidance aspects of psychological inflexibility breed maladaptive tendencies and consequently affect psychological health and QoL. This highlights the mediating role of AC. Our findings show that psychological inflexibility was strongly and positively associated with avoidance coping; affirming that inflexibility breeds avoidance tendencies and thought suppression [26, 27, 43]. These deter positive coping [28]. In this direction, avoidance coping is expected to lower mental health and QoL. On the contrary, our findings indicate that although associated with poor mental health, avoidance coping was positively associated with QoL during the COVID-19 pandemic. This suggests that whereas avoidance coping has negative consequences for mental health and wellbeing in the long term, it does provide temporary relief in the short term in highly distressing situations.

The study also considered the perceived threat of COVID-19, adherence to COVID-19 control measures, and general mental health as further mediators of the effects of psychological inflexibility and avoidance coping on QoL. Both psychological inflexibility and avoidance coping were negatively associated with the perception of the threat of COVID-19 and adherence to control measures. The possible explanation is that psychological inflexibility and avoidance coping result in underestimation of the threat posed by a distressing situation. The devaluation of threats leads to non-adherence to preventive measures or guidelines. Previous research has highlighted threat perceptions’ role on adherence [37, 88]. One of the contributions of the present study is the discovery that psychological inflexibility and avoidance coping influence the level of perceived threat, which may consequently lead to non-adherence.

Interestingly, both perceived threat and adherence to COVID-19 control measures were negatively related to the QoL of refugees. The perceived threat level could imply that individuals experience more intense psychological distress, which consequently influences the ratings of one’s own QoL [36, 38, 88, 89]. Concerning the negative association between adherence to COVID-19 control measures and QoL, it would be expected that adherence leads to a better QoL [64]. However, with the unprecedented outbreak of COVID-19, the restrictions to control it and the enforcement of these restrictions were rather drastic. Therefore, the level of adjustments in the way of living was also intense in a very short time. Consequences of this rapid change in how people live have been reported, including worsening of mental health problems and gender-based violence [90]. The challenge is even greater for refugees, especially the newly arrived, who may still have problems adjusting to the new environment and the new social and cultural realities, which have further implications for their wellbeing [91].

The findings supported the proposed serial mediation model. All seven mediation paths were significant, implying that the effects of psychological inflexibility on QoL were mediated by avoidance coping and further by the perceived threat of COVID-19, adherence to COVID-19 control measures, and general mental health. This reaffirms psychological inflexibility as an important factor influencing coping; whether one avoids or deals with the problem ultimately influences mental health and QoL [28, 30]. In crises and disasters such as the COVID-19 pandemic, the findings indicate that psychological inflexibility and the associated avoidance coping strategy are essential determinants of how individuals appraise the threat posed by the crisis and whether they adhere to or ignore the control guidelines. Whereas avoidance coping seems to help refugees to achieve a good level of psychological comfort in the short term during a crisis, there is need to investigate the long-term implications.

### Practical implications

The present study is one of the few that have attempted to provide an understanding of how refugees coped and adhered to the COVID-19 restrictions, especially in resource-constrained refugee settlements in less developed countries. In policy terms, our findings suggest that in further efforts to end the pandemic, specific interventions should be tailored to the needs of refugees and the context of refugee settlements. Interventions and communications that target the general public tend to be less effective for refugees given the language barrier, cultural differences, and existing mental health problems arising from living contexts and past traumatic experiences. There is also a need for policy incorporating psychosocial components in crisis interventions targeting refugees. This is critical in improving psychological health and quality of life during times of crisis.

In practical terms, our results suggest that psychological flexibility/inflexibility is a big determinant of people’s coping mechanisms when responding to unprecedented crises requiring immediate behavioral change. Support interventions such as mindfulness and acceptance-based training that promote psychological flexibility must be intensified [30, 67, 92, 93]. Behavioral and cognitive skills promoted by such interventions should be incorporated into programs when designing health-related communication to control the spread of pandemics and related crises. For refugee populations, the core principles of ACT that promote psychological flexibility can be adopted in routine psychotherapy and psychosocial interventions. This can be useful in improving their integration and wellbeing even in the absence of health or other related crises.

Moreover, there are still efforts to bring the COVID-19 pandemic to an end. Extant research indicates that non-pharmaceutical interventions are critical to the effort towards ending the COVID-19 pandemic [94]. Even after the advent of COVID vaccines, behavioral interventions remain necessary given that vaccination uptake is also behavioral. It also demands adherence. Moreover, the isolated incidences of the resurgence in COVID-19 cases show that more efforts are still required to end the pandemic. Our findings contribute to understanding psychological variables that are relevant to behavioral adjustments by individuals towards ending the pandemic. For example, reducing psychological inflexibility could positively change vaccine uptake. Low psychological flexibility is essential for mental health [25, 95]. Therefore, interventions to reduce psychological inflexibility could also be useful in overcoming refugees’ existing or emerging mental health problems.

### Limitations

Our study has some limitations that should be considered when generalizing findings. First, the study was conducted in two settlements out of the fourteen established refugee settlements in Uganda. Whereas these are among those hosting the largest numbers of refugees, they primarily host refugees from South Sudan and Somalia. Hence the representativeness of these populations could be a major limitation when applying our findings. Nonetheless, the results apply to at least two of the largest refugee groups in the country. Second, we translated the questionnaire into Arabic and Somali languages using the back-translation procedure. However, we did not conduct validation studies from the translated instruments. Future studies could consider adaptation of psychological scales and questionnaires to the context of refugee populations. Third, we tested our hypotheses using cross-sectional data obtained using self-report instruments. Therefore, common methods bias cannot be ruled out. Moreover, some associations, for example, between avoidance coping and mental health as well as QoL can change from positive in the short term to negative in the long term. Hence longitudinal studies would be useful in making causal conclusions about these relationships and the long-term impact of psychological inflexibility and coping on refugees’ mental health and quality of life.

## Conclusion

The study investigated a serial mediation model of effects of psychological inflexibility on quality of life of refugees in Uganda during the COVID-19 pandemic. Our findings reveal psychological inflexibility was associated with avoidance coping, lowered perceptions of the threat posed by COVID-19, and mental health. Consequently, psychological inflexibility was negatively associated with refugees’ quality of life during the pandemic. Furthermore, process analysis revealed that psychological inflexibility as a psychological attribute associated with avoidance might lead individuals into maladaptive coping behaviors, which consequently influenced refugees’ perceptions of the magnitude of threat posed by COVID-19, the level of adherence to control measures, and lowering mental health. Therefore, incorporating interventions to enhance psychological flexibility and reduce inflexibility would be important when designing disease control measures and future behavioral change interventions to promote mental health and QoL in refugee populations. However, it might also be important to all pay attention to the impact of gender differences and differences among the types of settlements. The study reveals that these factors are significantly associated with avoidance coping and quality of life.

## Supporting information

S1 Data SPSS file. This data file will also be available on Dryad: https://doi.org/10.5061/dryad.zs7h44jg5.
(RAR)
